# Chondroitin Sulfate-Based Imatinib Nanoparticles Targeting Activated Hepatic Stellate Cells Against Hepatic Fibrosis

**DOI:** 10.3390/pharmaceutics17030351

**Published:** 2025-03-09

**Authors:** Xunzhi Liu, Changlong Fang, Hongling Yu, Lu Huang, Jiaxing Feng, Shiqin Luo, Li Song, Mengying Wu, Yulu Tan, Jianxia Dong, Tao Gong, Peihong Xiao

**Affiliations:** 1 Key Laboratory of Drug-Targeting and Drug Delivery System of the Education Ministry and Sichuan Province, Sichuan Engineering Laboratory for Plant-Sourced Drug and Sichuan Research Center for Drug Precision Industrial Technology, West China School of Pharmacy, Sichuan University, Chengdu 610041, China; liuxunzhi00@163.com (X.L.); 17844613439@163.com (H.Y.); huanglu66666@126.com (L.H.); f1017484487@163.com (J.F.); luoshiqin1999@163.com (S.L.); wumengying15@163.com (M.W.); yulutan_scu_edu@163.com (Y.T.); gongtaoy@126.com (T.G.); 2 Department of Pharmacy, Chongqing University Fuling Hospital, Chongqing University, Chongqing 408099, China; fangchanglong12@163.com; 3 Department of Laboratory Medicine and Sichuan Provincial Key Laboratory for Human Disease Gene Study, Sichuan Provincial People’s Hospital, School of Medicine, University of Electronic Science and Technology of China, Chengdu 610041, China; 202421130234@std.uestc.edu.cn; 4 Department of Pharmacy, West China Hospital, Sichuan University, Chengdu 610041, China; 15802891207@163.com

**Keywords:** 1-hexadecamine modified chondroitin sulfate, CD44 receptors, activated hepatic stellate cells, liver fibrosis, imatinib

## Abstract

**Background**: Activated hepatic stellate cells (aHSCs) play a significant role during the onset of hepatic fibrosis, ultimately leading to excessive deposition of extracellular matrix (ECM) and other typical pathological features, and thus have become a popular target for the treatment of hepatic fibrosis. However, current aHSC-centric therapy strategies achieve unsatisfactory results, mainly due to the lack of approved anti-fibrosis drugs and sufficiently efficient aHSC-targeted delivery systems. In this study, our aim was to develop an Imatinib-loaded nanoparticle delivery system based on a chondroitin sulfate derivative to enhance aHSC targeting efficiency, improve the therapeutic effect for hepatic fibrosis, and investigate the underlying mechanism. **Methods**: The carboxyl group of chondroitin sulfate and the amino group of 1-hexadecylamine were linked by an amide bond in this study to produce the amphiphilic carrier CS-HDA. Then, the Imatinib-loaded nanoparticles (IM-CS NPs) were designed to efficiently target aHSCs through CD44-mediated endocytosis and effectively inhibit HSC overactivation via PDGF and TGF-β signaling pathways. **Results**: Both in vitro cellular uptake experiments and in vivo distribution experiments demonstrated that CS-HDA-modified nanoparticles (IM-CS NPs) exhibited a better targeting ability for aHSCs, which were subsequently utilized to treat carbon tetrachloride-induced hepatic fibrosis mouse models. Finally, significant fibrosis resolution was observed in the carbon tetrachloride-induced hepatic fibrosis mouse models after tail vein injection of the IM-CS NPs, along with their outstanding biocompatibility and biological safety. **Conclusions**: IM-loaded NPs based on an amphiphilic CS derivative have remarkable antifibrotic effects, providing a promising avenue for the clinical treatment of advanced hepatic fibrosis.

## 1. Introduction

Hepatic fibrosis, recognized as an inevitable pathological process characterized by abnormal proliferation of connective tissue in the liver caused by various pathogenic factors in the course of chronic liver disease, poses a serious threat to human health worldwide, with more than 2 million deaths annually [1,2,3]. Previous studies have proven that the release of inflammation-related chemokines, such as transforming growth factor beta (TGF-β) and platelet-derived growth factor (PDGF), can lead to the activation and proliferation of hepatic stellate cells (HSCs) [4,5,6,7,8,9]. As collagen is excessively secreted and expressed by fibroblasts, extracellular matrix (ECM), especially type I and III collagen, is deposited excessively, resulting in a chronic inflammatory response [10,11]. Without timely intervention or effective treatment, hepatic fibrosis will gradually develop into life-threatening cirrhosis, liver failure, and even liver cancer [12,13,14]. The activation of HSCs has been proven to be the core event during the onset of hepatic fibrosis; thus, aHSC-centric therapy has become a popular treatment strategy for hepatic fibrosis [15]. Unfortunately, reported studies that aimed at decreasing aHSC production or transforming aHSCs to a quiescent state obtained unsatisfactory results, as liver sinusoidal endothelial cells (LSECs) act as blood–HSC barriers, leading to the low targeting efficiency of nanoformulations toward aHSCs [16,17]. Therefore, there is an urgent need to develop a more effective anti-fibrotic therapy based on aHSC targeting.

The lack of appropriate drugs is one of the major reasons for the poor outcome of anti-fibrotic treatment. Although many small molecules are currently undergoing clinical trials, none has been approved for hepatic fibrosis therapy due to the complex pathogenesis, poor tissue-targeting ability, and low drug concentrations at lesion sites. The “new use of old drugs” may be a potentially useful approach for treating liver fibrosis, as the safety of the drugs used can withstand scrutiny. Imatinib (IM), a novel tyrosine kinase inhibitor, can selectively inhibit two key signaling pathways in HSC activation and fibrogenesis, namely, the PDGF and TGF-β pathways, the latter being considered the strongest known inducer of hepatic fibrogenesis [18,19,20,21]. Thus, it is expected to be a candidate drug for the treatment of hepatic fibrosis [22]. However, the current marketed formulations of IM are only oral tablets and capsules, and conventional IM therapy suffers from low target-tissue concentrations and increased toxicity to other tissues (especially the skin, lungs, and heart) [23]. Therefore, developing a suitable targeting drug delivery system (TDDS) to increase the concentration of IM in aHSCs and reduce the toxicity to other cells emerges as a promising strategy for improving its therapeutic efficacy in hepatic fibrosis treatment.

Chondroitin sulfate (CS), a major component of the ECM, is widely distributed in human and animal cartilage tissues [24,25,26]. As a natural macromolecule and human endogenous substance, CS is an ideal carrier for IM due to not only its excellent biocompatibility, but also its superior aHSC-targeting ability, which is ascribed to its ability to bind to the CD44 receptors overexpressed on aHSCs [27,28,29,30,31]. Although CS has a good binding affinity with aHSCs, the extremely hydrophilic nature of CS itself is detrimental to the delivery of small-molecule drugs. The previous pioneering studies from Professor Gong’s research group have confirmed that the amphiphilic CS not only exhibits better drug loading efficiency but also demonstrates superior performance in aHSC-targeting ability [5]. Inspired by this, we speculate that a well-orchestrated targeting delivery system of IM constructed on the basis of an amphiphilic CS derivative might greatly improve the therapeutic efficacy of hepatic fibrosis.

**Scheme 1:**
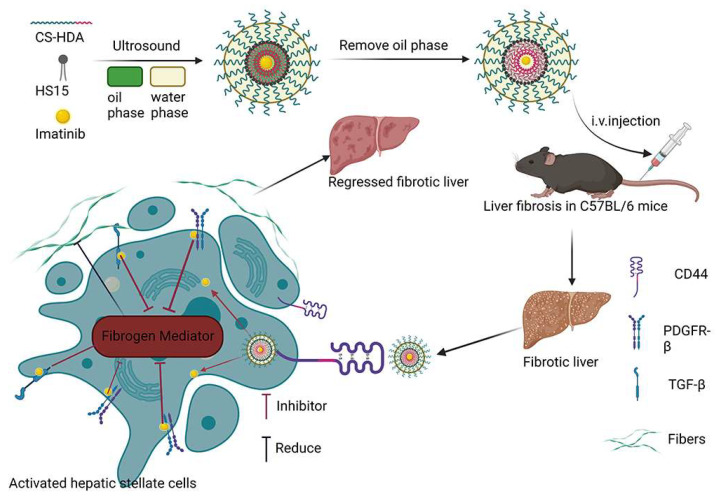
Strategic illustration for the application of CS nanoparticles to target aHSCs.

Herein, we propose an Imatinib-loaded drug delivery system based on an amphiphilic CS derivative for improving the target delivery efficiency to aHSCs to effectively treat hepatic fibrosis. Specifically, we first synthesized amphiphilic chondroitin sulfate-1-hexadecylamine (CS-HDA) conjugates and then constructed IM-CS nanoparticles (IM-CS NPs) using the emulsified solvent volatilization method. The lipophilic side chains of CS-HDA are inserted into the hydrophobic cores of the nanoparticles through intermolecular hydrophobic interactions, leading to an improved stability of the NPs, while the hydrophilic main chains on the surfaces of the NPs acts as the targeting ligands to aHSCs via CD44 receptor-mediated endocytosis. To further improve the targeting delivery efficiency of the nanoformulations to aHSCs, the particle sizes of IM-CS NPs were controlled in the range of 85–95 nm by prescription screening. Scheme 1 illustrates the strategic design of the IM-CS NPs, showing the targeting mechanism toward activated hepatic stellate cells (aHSCs), along with their application in anti-hepatic fibrosis therapy. We first evaluated the uptake levels of IM-CS NPs by various cells using fluorescence labeling methods and investigated the aHSC-targeting efficacy of IM-CS NPs in vitro. Then, experiments on pharmacokinetics and in vivo distribution and the validation of aHSC-targeting delivery were performed using normal mice and carbon tetrachloride-induced liver fibrosis mice. Finally, the liver fibrosis mice and normal mice were used to evaluate the anti-fibrotic efficacy and conduct a preliminary safety assessment of IM-CS NPs.

## 2. Materials and Methods

### 2.1. Materials

Imatinib (IM; Meilun, Dalian, China), Chondroitin Sulfate (CS; Meilun, Dalian, China), 1-Hexadecylamine (HDA; J&K Scientific, Beijing, China), Sulfo-NHS (Macklin, Shanghai, China), EDCI (Aladdin, Shanghai, China), 1,1′-dioctadecyl-3,3,3′,3′-tetramethylindodicarbocyanine perchlorate (DID; Biotium, Hayward, CA), DAPI (Beyotime, Shanghai, China), α-SMA antibody, (HuaBio, Hanzhou, China), type I collagen antibody (HuaBio, Hanzhou, China), CD44 antibody (HuaBio, Hanzhou, China), F4/80 antibody, CD31 antibody, HNF-4α antibody, (Abcom, America), CCK-8 (Biosharp, Hefei, China), Middle Chain Triglyceride (MCT; Yuanyebio, Shanghai, China), and 12-Hydroxy-octadecanoicacid polymer with α-hydro-ω-hydroxypoly(oxy-1,2-ethanediyl) (HS15, BASF, Ludwigshafen, Germany) were used in this study.

### 2.2. Synthesis of Chondroitin Sulfate-1-Hexadecylamine (CS-HDA) Conjugates

1-Hexadecylamine-modified CS was synthesized by amide reaction. Briefly, 9.56 mg of chondroitin sulfate was dissolved in ultrapure water (1.5 mL). Then, 0.04 mol of EDC and 0.04 mol of Sulfo-NHS were added to the chondroitin sulfate solution as catalysts, and they were stirred until fully dissolved. Next, 0.02 mol of HDA was dissolved in 3 mL NMP and the dissolution was accelerated by heating in a water bath, and when it was fully dissolved, it was slowly added dropwise to the chondroitin sulfate solution. The reactants were then stirred continuously at 25 °C for one day. Finally, the reaction mixture was precipitated in pre-cooled acetone at 4 °C, while the impurities and by-products were removed by dialysis using different ratios of ethanol and ultrapure water for more than 96 h. A 50% water–50% ethanol mixed solution was used initially, followed by pure water. The white flocculent product, CS-HDA, was obtained by freeze-drying after centrifugation (10,000 rpm, 10 min) to remove insoluble impurities.

### 2.3. Characterization of CS-HDA

^1^H NMR spectra of CS-HDA were acquired on a Varian spectrometer (FT-80A; Varian, CA, USA). CS-HDA and CS were dissolved in D_2_O. The degree of modification of the novel synthetic material CS-HDA was determined by calculating the ratios of the peak areas of the characteristic groups present in the spectra.

### 2.4. Preparation and Characterization of CS NPs

#### 2.4.1. Construction of CS NPs

The IM-CS NPs were prepared as follows: 60 mg of HS15 and 10 mg of CS-HDA were added to 4 mL of distilled water. Then, 4 mg of IM and 15 mg of MCT were added to 0.4 mL of organic solvent, and after both were fully dissolved, chloroform was slowly dropped into the aqueous solution. Finally, the mixture was sonicated at 180 w for 6 min and then evaporated by vacuum spinning at 37 °C for 15 min to obtain IM-CS NPs with a drug content of 1 mg/mL. Meanwhile, conventional drug-loaded nanoparticles (IM NPs) were prepared under the same conditions without the addition of CS-HDA, serving as controls for subsequent experiments.

In the subsequent experiments, the fluorescently labeled preparations (DID NPs and DID-CS NPs) were prepared exactly as the IM NPs/IM-CS NPs were prepared, except that DID was used instead of IM.

#### 2.4.2. Critical Micelle Concentration of CS-HDA with Different Degrees of Modification and HS-15 Mixed Materials 

By using fluorescent probes, the CMCs of mixed micelles of HS15 and CS-HDA with different substitution degrees were evaluated by fluorescence spectroscopy. In summary, a 0.1-mL solution of tetrahydrofuran in pyrene was prepared and subsequently diluted into 5-mL volumetric flasks, yielding a concentration of 4.0 × 10^−3^ mol/L. After the flasks were left to allow the tetrahydrofuran to evaporate in a fume hood, 5 mL quantities of mixed micellar solutions of HS15 with different concentrations (1.0 × 10^−4^ to 1.0 mg/mL) of CS-HDA substitutions were added. After sonication for 30 min to dissolve the mixtures well, the volumetric flasks were incubated at 37 °C for 1 day. The measurement conditions for I_1_ and I_3_ fluorescence values were Ex = 336 nm/Em = 373 nm and Ex = 336 nm/Em = 383 nm, respectively. The concentration of mixed micelles (C) was plotted by I_3_/I_1_ and fitted by GraphPad Prism 8.0.1, and the concentration corresponding to the abrupt change in the curve was the CMC value.

#### 2.4.3. Characterization of CS NPs

The particle sizes and polydispersive indexes (PDIs) of the IM-CS NPs and IM NPs were measured by a Malvern Zetasizer Nano ZS90. The morphology of the NPs was examined by a Hitachi H-600 TEM (Hitachi, Ibaraki Prefecture, Japan). The sample preparation method was as follows: the diluted IM-CS NPs and IM NPs were dropped onto a copper grid, negatively stained with 2% phosphotungstic acid, and appropriately dried.

In addition, the encapsulation efficiency (EE) and drug loading capacity (DLC) are two of the most important physicochemical indicators of TDDS. These two indicators were measured by ultrafiltration and calculated according to Equations (1) and (2):

(1) $$\mathrm{EE}\;(\%,\;w/w)=\frac{Weight\;of\;drug\;encapsulated}{Weight\;of\;drug\;added}\times 100\%$$

(2) $$\mathrm{DLC}\;(\%,\;w/w)=\frac{Weight\;of\;drug\;encapsulated}{Weight\;of\;nanoparticles}\times 100\%$$

The specific method of ultrafiltration is as follows: Place 400 µL of drug-loaded nanoparticles into the inner tube of an ultrafiltration tube with a molecular weight cutoff of 30 kDa and centrifuge. Collect 200 µL from the outer tube, disrupt the emulsion with methanol, and dilute to 2 mL. After centrifugation, sample the solution and measure the amount of encapsulated drug using HPLC. Additionally, take 200 µL of untreated nanoemulsion, disrupt the emulsion with methanol, dilute to 2 mL, and measure the amount of added drug. And the HPLC conditions are as follows: column: C-18 (250 × 4.6 mm, 5 µm); mobile phase: 0.1% phosphoric acid aqueous solution–acetonitrile = 20:80 (*v*/*v*); flow rate: 0.8 mL/min; column temperature: 30 °C; detection wavelength: 258 nm; injection volume: 20 µL

In this study, the storage, serum, and dilution stability of the IM NPs/IM-CS NPs were evaluated through three experiments as follows: (1) nanoparticles (1 mg/mL) were stored in distilled water at 4 and 37 °C for 5 consecutive days; (2) nanoparticles and serum (FBS, Gibco, USA) were mixed at a volume ratio of 1:1 and incubated at 37 °C for 48 consecutive hours; (3) nanoparticles were diluted with distilled water at ratios of 5×, 10×, 20×, 35×, and 50× (*v*/*v*). The stability of the nanoparticles was assessed by periodically measuring their particle size.

### 2.5. In Vitro Release of IM-CS NPs

The release behavior of the IM-CS NPs was measured by HPLC in vitro. Briefly, 8000 D dialysis bags were filled separately with 1 mL of Imatinib Solution, 1 mL of IM NPs, and 1 mL of IM-CS NPs (all concentrations were 1 mg/mL). Next, the bags were placed in 50 mL of release medium and set at a constant temperature and placed in a speed shaker at 37 °C and 100 rpm. The release medium was 0.1 M phosphate buffer solution (pH = 7.4) containing 0.2% Tween 80. At each preset time point, 1.0 mL of old medium was removed from the container and immediately replenished with 1.0 mL of new medium. Finally, the samples were analyzed by HPLC (Agilent G1311C; Agilent Technologies, CA, USA).

### 2.6. Cells and Animals

HSC-T6, HepG_2_, RAW_264.7_, and HUVEC cells were obtained from the Chinese Academy of Science Cell Bank. HSC-T6, HepG_2_, and RAW_264.7_ cells were incubated with DMEM, while HUVEC cells were incubated with RPMI 1640. All media contained 10% FBS, 100 U mL^−1^ streptomycin, and 100 U mL^−1^ penicillin.

Male C57BL/6J mice (6 weeks old) and male SD rats (180~220 g) were purchased from Chengdu Dossy Biotechnology Co., Ltd. (Chengdu, China). All the animal protocols and experiments were conducted in accordance with the ARRIVE guidelines, and all the animal experimental operations complied with the requirements of the National Act on the Use of Experimental Animals (China) and were approved by the Animal Ethics Committee of Sichuan University.

### 2.7. Cellular Study

#### 2.7.1. Activation of HSCs

HSC-T6 cells were incubated at a seeding density of 1 × 10^5^ cells/well for 12 h and then continued to be incubated with different concentrations of TGF-β, and the concentrations of TGF-β1 were 0, 2, 5, and 10 µg/mL. After 24 h, the cell samples were analyzed by WB to characterize the expression levels of α-SMA and type I collagen in the cells. The specific operation of WB was as follows: Proteins were separated by 10% SDS-PAGE electrophoresis and transferred to a 0.45 µm PVDF membrane. The membrane was blocked with 5% skim milk at room temperature for 1 h, followed by incubation with primary antibodies (α-SMA, type I collagen, and GAPDH) in a shaker at 4 °C overnight. Afterward, HRP-conjugated IgG was added and incubated at room temperature for 1 h. Finally, ECL luminescence was performed, and the results were visualized.

#### 2.7.2. CD44 Expression Levels

Activated HSC-T6, HepG_2_, and RAW_264.7_ cells were incubated at a density of 1 × 10^5^ cells/well for 24 h. Cell lysate samples were collected for analysis of cellular CD44 expression levels by WB.

#### 2.7.3. Cellular Uptake

Activated HSC-T6, HepG_2_, and RAW_264.7_ cells (1 × 10^5^ cells per well) were evenly spread in 12-well plates for 24 h. After discarding the old medium and washing with PBS, 1 mL of DMEM incomplete medium with DID Solution, DID NPs, and DID-CS NPs (final DID concentration was 300 ng mL^−1^) was added to the respective cell wells. After treatment, the cells continued to be incubated for another 2 h, after which the cells were trypsinized and the cell precipitate was collected. The cell precipitate was washed with PBS to remove excess fluorescent material from the cell surface, followed by resuspension of the cells in 0.3 mL of PBS. Finally, the fluorescence intensity of each group of samples was measured by an FACS Celesta flow cytometer (BD, CA, USA).

#### 2.7.4. Inhibition of Cellular Uptake

To study the cell entry mechanism of DID-CS NPs, we first pretreated the activated HSC-T6 cells for 1 h with specific inhibitors, then continued to incubate the DID-CS NPs and measured their uptake efficiency. The dosages of uptake inhibitors are shown in Table 1. Briefly, activated HSC-T6 cells were pretreated with different inhibitors at 37 °C or incubated without any inhibitors at 4 °C. After discarding the old medium and washing with PBS, 1 mL of DMEM incomplete medium with DID-CS NPs (final DID concentration was 300 ng mL^−1^) was added to the cell wells. After the cells continued to be cultured for 4 h, the cells were trypsinized and the cell precipitate was collected. The cell precipitate was washed with PBS to remove excess fluorescent material from the cell surface, followed by resuspension of the cells in 0.3 mL of PBS. Finally, the fluorescence intensity of each group of samples was measured. The uptake efficiency of the cells was characterized according to Equation (3):

(3) $$\mathrm{Relative}\;\mathrm{uptake}\;(\%)=\frac{Fluorescence\;intensity\;of\;cells\;after\;treatment}{Fluorescence\;intensity\;of\;cells\;without\;treatment}\times 100\%$$

#### 2.7.5. Cytotoxicity Assay

In vitro cytotoxicity was assessed by CCK-8 assay. Activated HSC-T6 and HUVEC cells were cultured at a seeding density of 1 × 10^4^ cells/well for 24 h. The original medium was discarded, and incomplete medium containing corresponding concentrations of IM solution, IM NPs, or IM-CS NPs was added, followed by continued incubation for 24 h. The concentration gradient of IM was as follows: 0, 0.5, 1, 2, 4, 8, 16, 32 µg mL^−1^. After 24 h of incubation, the old medium was discarded and 100 µL of 10% CCK-8 solution (incomplete medium dilution) was added to each well, and incubation continued for 4 h. The absorbance of each well was measured separately, then the cell viability was calculated according to Equation (4):

(4) $$\mathrm{Cell}\;\mathrm{viability}\;(\%)=\frac{Absorbance\;of\;cells\;after\;treatment}{Absorbance\;of\;cells\;without\;treatment}$$

#### 2.7.6. Expression Levels of Type I Collagenase

Following a 24 h incubation period of activated HSC-T6 cells at a density of 1 × 10^5^ cells/well, the original medium was discarded and 1 mL of blank medium or medium containing IM (IM solution, IM NPs, or IM-CS NPs) was added. The IM dosing concentration was 0.5 µg/mL. Another group of HSC-T6 cells with neither TGF-β1 stimulation nor IM was set up as a control group. Cells continued to be incubated for 12 h and were then pretreated for immunofluorescence staining. Finally, immunofluorescence staining was performed to characterize the expression of type I collagen. The details of the staining were as follows: Cells were fixed with pre-cooled 4% paraformaldehyde fixative, permeabilized by incubation with 0.1% Triton X-100 at room temperature, and blocked with 5% goat serum at room temperature for 1 h. The cells were then incubated with primary antibody (collagen type I high-affinity purified IgG, 1:200 dilution) overnight at 4 °C, followed by incubation with secondary antibody (FITC-conjugated IgG, 1:200 dilution) at room temperature for 1 h.

### 2.8. Establishment of Liver Fibrosis Mouse Model

In this study, male mice were selected as model mice. We induced liver fibrosis in the mice by intraperitoneal injection of 20% CCl_4_ (olive oil dilution). The carbon tetrachloride dose was calculated at 3 mL/kg of body weight, based on the weight of each mouse, and was administered twice a week for a period of 4 weeks. Changes in mouse body weight, behavior, and liver appearance were observed to aid the assessment of liver fibrosis modeling. Liver H&E, Masson, and Sirius Red staining were performed to evaluate whether the liver fibrosis mouse model had been successfully established. The staining details were as follows:

H&E Staining: Following deparaffinization and rehydration through a graded ethanol series (absolute ethanol for 5 min, 95% ethanol for 5 min, 85% ethanol for 5 min, 75% ethanol for 5 min, distilled water rinse for 1 min), sections were stained with Harris hematoxylin for 4 min, rinsed with tap water, differentiated in 0.8% hydrochloric acid–alcohol (2 sec), blued in lithium carbonate solution (optional), counterstained with eosin (20 sec), dehydrated through 95% ethanol (5 min) and absolute ethanol (two changes, 2 min each), cleared in xylene, and mounted with neutral balsam.

Masson Staining: After deparaffinization/rehydration (as above), sections were fixed in Bouin’s or Zenker’s solution overnight, rinsed, stained with iron hematoxylin (5–10 min), differentiated in 0.8–1% hydrochloric acid–alcohol, blued in tap water, stained with Ponceau-acid fuchsin (5–10 min), treated with phosphomolybdic acid (5 min), counterstained with aniline blue (5 min), differentiated in 1% glacial acetic acid (1 min), dehydrated through graded ethanol (95% ethanol, multiple changes), cleared in xylene, and mounted for collagen visualization.

Sirius Red Staining: Deparaffinized/rehydrated sections were stained with celestine blue (5–10 min) or Harris hematoxylin, rinsed, immersed in saturated Picro-Sirius Red solution (15–30 min), dehydrated through four changes of absolute ethanol, cleared in xylene, and mounted with neutral balsam. Collagen deposition was assessed microscopically under polarized light.

### 2.9. Biodistribution

To compare the differences in the distribution of IM NPs and IM-CS NPs in the liver fibrosis mice, the mice were randomly divided into three groups (*n* = 12). DID Solution, DID NPs, and DID-CS NPs were administered at a DID dose of 200 µg kg^−1^, respectively. At 2, 6, 12, and 24 h after administration, three mice in each group were sacrificed, and then the heart, liver, spleen, lungs, and kidneys were excised. The above isolated organs were washed with PBS, and ex vivo imaging of fluorescence was performed with an IVIS Lumina ll (PerkinElmer, Waltham, MA, USA).

### 2.10. Validation of IM-CS NPs Targeting aHSCs via CD44 Receptors

#### 2.10.1. Validation of Co-Localization Between aHSCs and CD44 Receptors

Co-localization of aHSCs and CD44 receptors was verified by immunofluorescence staining assay. Liver tissue from mice with liver fibrosis was prepared in frozen sections. The activated HSCs were detected using an α-SMA antibody, while Kupffer cells were identified through labelling with an F4/80 antibody. Furthermore, HSECs were labelled with a CD31 antibody, and HPCs were labelled with an HNF-4α antibody. Apart from these, CD44 receptors were labeled with CD44 antibody. The specific operations are as follows: Excise a small piece of liver tissue, embed it in OCT compound, prepare frozen sections at 8 µm thickness, and mount them on glass slides. Subsequently, wash the sections twice with PBS to remove embedding medium, then fix with 4% paraformaldehyde fixative for 10 min, followed by three PBS washes. Permeabilize cells with 0.2% Triton X-100 (diluted in PBS) at room temperature for 5 min, followed by two PBS washes. Block nonspecific antigens by incubation with goat serum (PBS-diluted) at room temperature for 1 h, then wash twice with PBS. Proceed with staining: Apply 50 µL primary antibody to cover tissue sections and incubate overnight at 4 °C. After three PBS washes (5 min each), incubate with Cy3-conjugated secondary antibody protected from light at room temperature for 1 h, followed by three PBS washes (5 min each). Counterstain nuclei with DAPI for 10 min and perform three PBS washes (5 min each). Finally, apply 100 µL anti-fade mounting medium; cover with a coverslip, avoiding air bubbles; seal the edges with colorless nail polish; and observe the fluorescence under a laser confocal microscope.

#### 2.10.2. Validation of Co-Localization of IM-CS NPs and CD44 Receptors

DID Solution, DID NPs, and DID-CS NPs were injected intravenously (i.v.) into normal mice and liver fibrosis mice at a DID dose of 200 µg kg^−1^. After 12 h, the mice were executed, and liver tissue was taken for frozen sections. CD44 receptors were labeled with FITC, and nuclei were labeled with DAPI.

### 2.11. Pharmacokinetics

Normal SD rats (180–200 g) were randomly divided into 3 groups (n = 5). Each group was injected with IM solution, IM NPs, and IM-CS NPs at an IM dose of 8 mg kg^−1^. Next, 300 µL of rat whole blood was collected through orbital blood sampling at 5, 15, 30, 60, 120, 240, 480, and 720 min post-injection. Finally, the IM concentration in plasma was determined by HPLC after pretreatment of plasma by organic solvent extraction. The details of organic solvent extraction are as follows: Take SD rat plasma and place it in centrifuge tubes. Add 10 volumes of ethyl acetate, followed by vigorous vortexing. After centrifugation at 10,000 rpm for 6 min, recover the upper organic layer. Repeat the extraction of the residue with another 10 volumes of ethyl acetate using the same method. Combine the organic layers from both extractions and evaporate to dryness using a nitrogen evaporator. Redissolve the residue in methanol, centrifuge to collect the supernatant, and filter it through a 0.22 µm microporous membrane prior to instrumental analysis.

### 2.12. Anti-Liver Fibrosis Study in Mice

Male C57BL/6J mice were randomly divided into five groups (*n* = 6): a healthy group, a saline group, an IM solution group, an IM NP group, and an IM-CS NP group. The healthy group was only fed normally, without any treatment. The remaining four groups were constructed by the aforementioned method, and after the successful construction of the liver fibrosis model, the mice were injected with saline, IM solution, IM NPs, and IM CS-NPs intravenously, and the dose of IM was calculated as 6 mg/kg. The dosing frequency was an average of 8 doses over 4 weeks. The mice were executed after the last administration of the drug. Body weight and liver weight were recorded. Liver appearance, H&E staining, Sirius Red staining, immunofluorescence staining, and immunohistochemistry staining analyses were performed to evaluate the treatment efficacy of IM-CS NPs. Meanwhile, some blood biochemical indicators in serum were measured.

### 2.13. Safety Evaluation

Normal male C57BL/6J mice were randomly divided into four groups (*n* = 5). These four groups were simultaneously injected with saline, IM solution, IM NPs, and IM CS-NPs twice a week at an IM dose of 6 mg kg^−1^, respectively. After 4 weeks, preliminary safety evaluations of IM-CS NPs by weight, blood count, H&E staining, and other analyses were performed.

### 2.14. Statistical Analysis

Results are presented as means ± SDs. One-way ANOVA was used to determine the significance of differences. Statistical significance was defined at * *p* < 0.05, ** *p* < 0.01, *** *p* < 0.001, and **** *p* < 0.0001.

## 3. Results

### 3.1. Synthesis of the Chondroitin Sulfate-1-Hexadecylamine (CS-HDA) Conjugate

**Figure 1:**
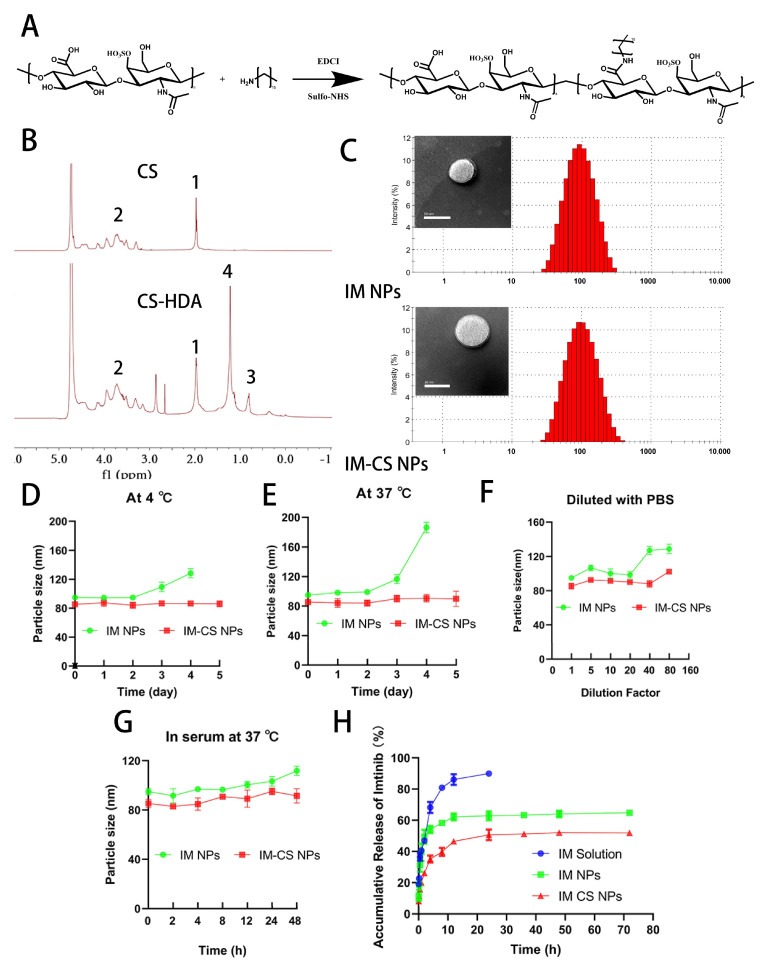
(**A**) Synthetic scheme of CS-HDA conjugates. The a represents the part that is not connected to HDA, and the b represents the part that is connected to HDA. (a+b=n) (**B**) ^1^H NMR spectra of CS and CS-HDA conjugate: characteristic peaks of CS (-COCH_3_-) and CS (sugar ring) appeared at 2.00 ppm (1) and 3.30–4.80 ppm (2); characteristic peaks of HAD (-CH_3_) and HDA (-(CH_2_) _n_-) appeared at 0.80 ppm (3) and 1.20 ppm (4). Characterization of NPs: (**C**) DLS size measurement and TEM images of IM NPs and IM-CS NPs. The stability of IM NPs and IM-CS NPs (**D**) at 4 °C, (**E**) at 37 °C, (**F**) diluted with PBS (pH = 7.4), and (**G**) in serum at 37 °C. Data represent means ± SDs (*n* = 3). (**H**) Imatinib release profiles of IM solution, IM NPs, and IM-CS NPs in PBS at pH = 7.4. Data represent means ± SDs (*n* = 3).

To prepare the amphiphilic CS-HDA conjugate, hydrophobic HDA was linked to hydrophilic chondroitin sulfate. As shown in Figure 1A, the amino group of HDA was attached to the carboxyl group of chondroitin sulfate through the amide reaction, which greatly improved the amphiphilicity of chondroitin sulfate, and its chemical mechanism was verified by ^1^H NMR. As shown in Figure 1B, the characteristic peaks of CS appeared at 2.00 ppm (-COCH_3_-) and 3.30–4.80 ppm (sugar ring). The characteristic peaks of HDA were found at 0.80 ppm (-CH_3_) and 1.20 ppm (-(CH_2_) _n_-) of the CS-HDA spectrum, indicating the successful attachment of HDA to the side chain of CS. The coupling degree of CS-HDA was quantified using the integral ratio between the hydrogen atoms of the acetyl peak (2.00 ppm, 3H, -COCH3-) in chitosan (CS) and the hydrogen atoms of the methyl peak (0.80 ppm, 3H, -CH3) in HAD (Figure S1). By altering the feed ratio of CS to HDA (while keeping the amounts of CS and the catalyst constant), we synthesized CS-HDA with varying degrees of substitution. The degree of modification of CS was determined by the ^1^H NMR hydrogen spectrum of CS-HDA. Finally, we synthesized CS-HDA with 10%, 20%, and 30% substitution degrees. The relationship between the corresponding feed ratios and the degrees of substitution is shown in Table S1.

### 3.2. Screening for Optimal CS-HDA Degree of Substitution

IM-CS NPs were synthesized via the emulsifying solvent evaporation method [32,33,34]. Using pyrene as a fluorescent probe to determine the CMC of mixed micelles through the fluorescence method, as shown in Figure S2A and Table S2, we can see that as the CS-HDA substitution degree increases, the CMC of the mixed micelles gradually decreases. We verified the targeting of aHSCs by CS-HDA with different degrees of substitution through cell uptake and distribution experiments in fibrosis mice. The experimental results in Figure S2B tell us that as the degree of substitution increased, activated HSC-T6 and HepG_2_ cells took up more DID-CS NPs. Similarly, as shown in Figure S2C, we obtained results with the same trend in distribution experiments in vivo. In summary, we chose CS-HDA with a degree of substitution of 30% to prepare IM-CS NPs for subsequent research.

### 3.3. Characterization of IM NPs and IM-CS NPs

The characteristics of the IM NPs and IM-CS NPs, including particle size, PDIs, DLC, and EE, are shown in Table 2. As shown in Figure 1C, the IM NPs and IM-CS NPs have a similar nanometer particle size, which indicates that the side chain of CS-HDA with a high degree of substitution can insert well into the hydrophobic core of the nanoparticle and has little effect on the particle size. TEM images tell us that the IM NPs and IM-CS NPs were almost spherical in shape and less than 80 nm in size. Such small particle sizes (<100 nm) ensure that the nanoparticles can more easily reach the aHSCs [35,36,37]. The DLC of the IM NPs was slightly higher than that of the IM-CS NPs, and the EEs of the IM NPs and IM-CS NPs were consistently above 97%. Additionally, the IM NPs and IM-CS NPs had good stability at (Figure 1D) 4 °C and (Figure 1E) 37 °C, (Figure 1F) diluted with PBS, and (Figure 1G) in serum, such that they were sufficient to meet the requirements of our subsequent experiments.

### 3.4. In Vitro Release Behavior of IM-CS NPs

Figure 1H shows the in vitro release profiles of the IM solution, IM NPs, and IM-CS NPs in PBS (pH = 7.4). By the overall release rates of the three formulations, there is no doubt that IM-CS NPs have the best release in vitro.

### 3.5. HSC-T6 Cells Were Successfully Activated and Exhibited High Expression of CD44 Receptors

TGF-β is one of the important cytokines that induces liver fibrosis, and it is involved in promoting the synthesis of ECM and inhibiting its degradation. Therefore, we chose TGF-β1 to activate HSC-T6 cells in this study [38,39]. After activation, HSCs can express α-SMA and synthesize and secrete ECM to participate in the formation of liver fibrosis. As shown in Figure S3A, under the stimulation of TGF-β1, we successfully activated HSC-T6. In the follow-up study, using TGF-β1, we chose 10 µg/mL TGF-β1 to complete this step.

CD44 receptors are involved in a variety of important physiological processes in cells. In the field of pharmacy, researchers have been inspired to use CD44 receptors as a target for cell-targeted drug delivery [40,41]. Investigating the positive expression rate of CD44 receptors in various cell types can make the design of targeted drug delivery systems more efficient and precise. As shown in Figure S3B, activated HSC-T6 and HepG_2_ cells highly express CD44 (CD44^+^), while RAW_264.7_ cells lowly express CD44 (CD44^−^). This lays a research foundation for designing a CD44-mediated targeted drug delivery system for aHSCs.

### 3.6. IM-CS NPs Can Be Selectively Taken Up by aHSCs

Most drugs work inside cells, so the key to successfully treating a disease is delivering a drug inside cells or even organelles [42,43]. In vitro cell experiments are an important method for studying targeted drug delivery systems [44,45,46]. To evaluate the IM-CS NP drug delivery system, we first wanted to study its delivery efficiency, mechanism of action, and targeting mechanism by an in vitro cellular assay.

**Figure 2:**
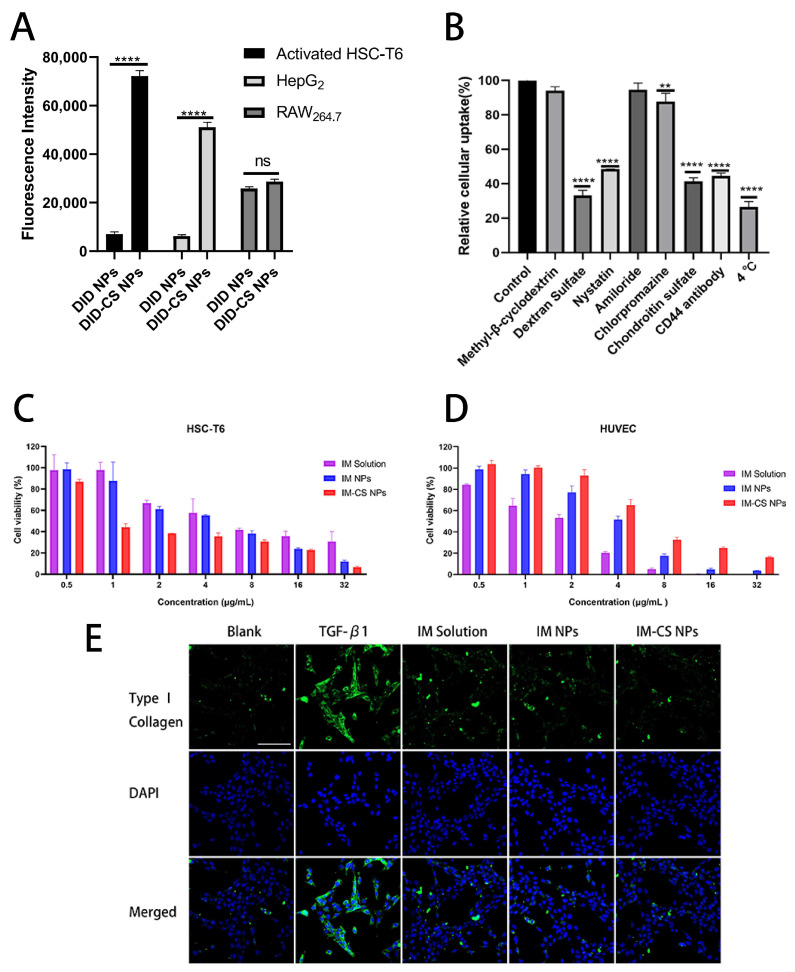
(**A**) Cellular uptake of DID NPs and DID-CS NPs after incubation with aHSCs (CD44^+^), HepG_2_ (CD44^+^), and RAW _264.7_ (CD44^−^) cells for 2 h. (**B**) Effect of reduced temperature (4 °C) and different inhibitors on uptake of IM from IM-CS NPs by activated HSC-T6 cells, observed by flow cytometric analysis (*n* = 3, means ± SDs). ** *p* < 0.01 and **** *p*<0.0001. Inhibitory effects of IM solution, IM NPs, and IM-CS NPs on the proliferation of (**C**) aHSCs and (**D**) HUVEC cells measured by the CCK-8 assay in vitro. Data represent means ± SDs (n = 6). Immunofluorescent staining of (**E**) type I collagen in HSC-T6 cells treated with DMEM and activated HSC-T6 cells treated with DMEM, IM solution, IM NPs, and IM-CS NPs. The scale bar represents 100 µm.

The experimental results in Figure 2A show that the fluorescence intensity of the DID-CS NP-treated activated HSC-T6 cells was almost ten times greater than that of those treated with DID NPs, and the HpeG_2_ cells showed a similar trend, while in Raw_264.7_ cells, the fluorescence intensity of Raw_264.7_ cells incubated with DID-CS NPs was only 1.1 times greater than that of those incubated with DID NPs. This strongly demonstrates that IM-CS NPs are more readily taken up by cells with a high expression of CD44 receptors. The fluorescence intensity of activated HSC-T6 cells incubated with DID-CS NPs was approximately two times higher than that of Raw_264.7_ cells, while DID NPs showed a completely opposite trends in the above two types of cells. Thus, DID-CS NPs can selectively accumulate in aHSCs while reducing their distribution in other cells.

In the cellular uptake inhibition assay, various endocytosis inhibitors reported in the literature were added to block or inhibit a certain endocytic pathway. DID-CS NPs were added after inhibition to investigate whether cellular uptake efficiency was affected. As shown in Figure 2B, preincubation at 4 °C triggered the most significant uptake reduction (73.6%), followed by dextran sulfate (66.8%), chondroitin sulfate (58.7%), CD44 antibody (55.6%), nystatin (51.5%), chlorpromazine (12.4%), MβCD (6%), and amiloride (5.5%). The uptake of DID-CS NPs in activated HSC-T6 cells incubated at 4 °C was almost completely inhibited, suggesting that the uptake of CS NPs is mainly energy-dependent active transport. Methyl-β-cyclodextrin and amiloride had no effect on the uptake of activated HSC-T6, while chondroitin sulfate, CD44 antibody, dextran sulfate, chlorpromazine, and nystatin significantly reduced the uptake of activated HSC-T6 cells. The results show that the main way of entry of DID NPs into aHSCs is energy-dependent endocytosis mediated by CD44, clathrin, and caveolin.

### 3.7. IM-CS NPs Selectively Kill aHSCs In Vitro

As mentioned above, activation of quiescent HSCs plays an important role in fibrosis [5,6,7]. Activated HSCs differentiate into a fibroblast phenotype and secrete substantial ECM [47,48,49]. Therefore, killing aHSCs is essential for the treatment of fibrosis. To investigate the cytotoxicity of CS NPs, we used the cytotoxicity assay to explore the killing effect of IM-CS NPs on aHSCs and HUVEC cells. Figure 2C tells us that the cell viability of the aHSCs treated with IM-CS NPs was much lower than that of the IM NP- or IM solution-treated groups at the equivalent drug concentrations. However, the exact opposite trend was observed in HUVEC cells (Figure 2D); it was clear that the IM solution-treated HUVEC cells had the lowest survival rate, while cytotoxicity was greatly attenuated in the IM NP- and IM-CS NP-treated HUVEC cells. The above results show that IM-CS NPs can selectively kill aHSCs in vitro and have a protective effect on normal cells such as HUVECs.

### 3.8. IM-CS NPs Inhibit Collagen Secretion from aHSCs

Collagen is vital in the life activities of animals and is involved in several physiological activities [50]. One of its important functions is its involvement in the formation of the ECM [51,52]. Under normal physiological conditions, its production and degradation are in a dynamic balance, but when this balance is disturbed, the overproduction of collagen leads to excessive deposition of ECM, which can lead to a variety of diseases, such as liver fibrosis [53,54,55,56]. Therefore, the detection of abnormal collagen is essential for the determination of the pathological conditions of the disease [57]. Figure 2E shows that after the IF staining experiments, the green fluorescence of type I collagen in the IM-CS NP treatment group was weaker than that in the IM NP and IM solution groups, demonstrating that the CS NPs reduced collagen secretion after targeted delivery of IM to aHSCs.

### 3.9. Establishment of Animal Models

The fibrosis model was constructed in accordance with the method detailed in Section 2.8 of this paper, as previously described [58,59]. As shown in Figure S3C, comparing the appearances of the livers, the surface of the livers of the CCl_4_-induced mice were uneven, hard, and dark pink, whereas the livers of the normal mice were smooth, soft and brittle, and dark red. The dissected liver tissues were processed for histological sectioning and staining. H&E stains showed that the CCl_4_-induced mice liver showed focal necrosis, moderate infiltration of inflammatory cells, fibrosis, hepatocyte edema, and fibrous encapsulation of regenerated hepatocytes. Masson and Sirius Red stains showed that CCl_4_-induced mouse livers had a large accumulation of collagen. These phenomena were not observed in the staining of tissue sections of normal mouse livers. The results showed that we successfully established a mouse model of liver fibrosis.

### 3.10. IM-CS NPs Prolong the Cycle Time of IM In Vivo

**Figure 3:**
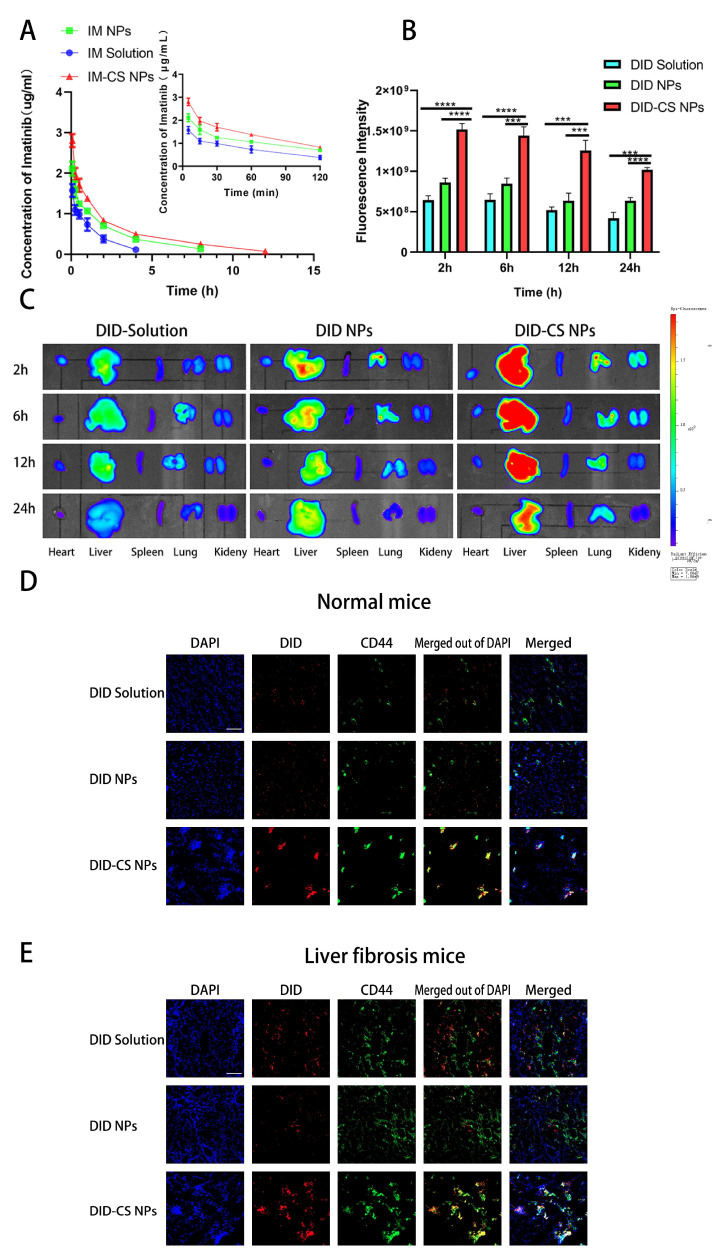
(**A**) In vivo pharmacokinetic profiles 0–12 h and 0–2 h after intravenous injection of IM solution, IM NPs, and IM-CS NPs in normal rats. Data represent means ± SDs (n = 5). The average fluorescence intensities of fibrosis model mice (**B**) at 2, 6, 12, and 24 h after injection of different preparations (means ± SDs, n = 3). *** *p* < 0.001; **** *p* < 0.0001. In vivo DID fluorescence images (**C**) show the biodistribution of DID solution, DID NPs, and DID-CS NPs in fibrosis livers at 2, 6, 12, and 24 h after injection. The immunolocalization of DID solution, DID NPs, and DID-CS NPs in (**D**) normal and (**E**) fibrosis livers. The cell nuclei were stained with DAPI (blue), CD44 receptors (aHSCs) were stained with FITC-labeled anti-mouse IgG (green), and DID fluorescence is displayed in red. The scale bar represents 200 µm.

Nanoparticles will be diluted by the blood after injection into the blood circulation, and this dilution will be accompanied by the nanoparticles being distributed to various organs (such as the liver, spleen, lungs, etc.), resulting in a dramatic reduction in the number of nanoparticles remaining in the blood [60]. Therefore, to increase the distribution of nanoparticles in liver tissues, we needed to examine whether the constructed nano-delivery system could prolong the time of IM in the blood circulation [61]. Figure 3A and Table 3 show the pharmacokinetic results. They tell us that the in vivo circulation time and area under the curve of the IM-CS NPs were higher than those of the IM solution and IM NPs, which lays a pharmacokinetic basis for the use of IM-CS NPs to treat liver fibrosis.

### 3.11. IM-CS NPs Selectively Accumulate in the Liver

The purpose of the TDDS is to selectively enrich the drug in the lesion site through a specific carrier, where the lesion site is what we usually call the target site [62]. The in vivo distribution experiment is an important method used to investigate targeted drug delivery systems. To better examine the liver targeting of IM-CS NPs, we examined the distribution of three groups of fluorescent agents (solution, NPs, and CS NPs) in fibrosis mice using IVIS. Figure 3B shows the semi-quantitative analysis of the in vivo distribution at the 2, 6, 12, and 24 h time points. The statistical plot of the experimental results proved that the liver fluorescence intensity in the CS NP-treated group was 1.76 and 2.35 times higher than that in the other two groups at 2 h, while the results were similar at 6, 12, and 24 h. IM-CS NPs have a significant fibrosis liver-targeting effect.

### 3.12. The Distribution of CD44 Receptors in Different Hepatocytes and the Co-Localization of CS NPs and aHSCs In Vivo

We used immunofluorescence staining to explore the expression of CD44 receptors in fibrosis livers. Activated HSCs were fluorescently labeled with α-SMA antibody, HPCs were fluorescently labeled with HNF-4α antibody, Kupffer cells were fluorescently labeled with F4/80 antibody, and HSECs were fluorescently labeled with CD31 antibody [63,64,65,66,67]. Figure S4 shows that CD44 receptors are mainly expressed on aHSCs and that a small amount are expressed on Kupffer cells.

We explored the selectivity of CS NPs for aHSCs by co-localization of DID with the CD44 receptors in each preparation. Figure 3D,E show that, in the DID-CS NP group, the red light and the green light almost completely overlap, and the red light is not distributed to areas other than those of the green light. It is shown that IM-CS NPs have excellent targeting to aHSCs and hardly distribute to other hepatocytes. In contrast, the DID solution and DID NPs were distributed to other sites than aHSCs. In conclusion, CS NPs have excellent targeting to aHSCs in vivo and can be used as an excellent targeting carrier for aHSCs with great potential for anti-liver fibrosis treatment.

### 3.13. IM-CS NPs Have Good Anti-Liver Fibrosis Efficacy at the Animal Level

**Figure 4:**
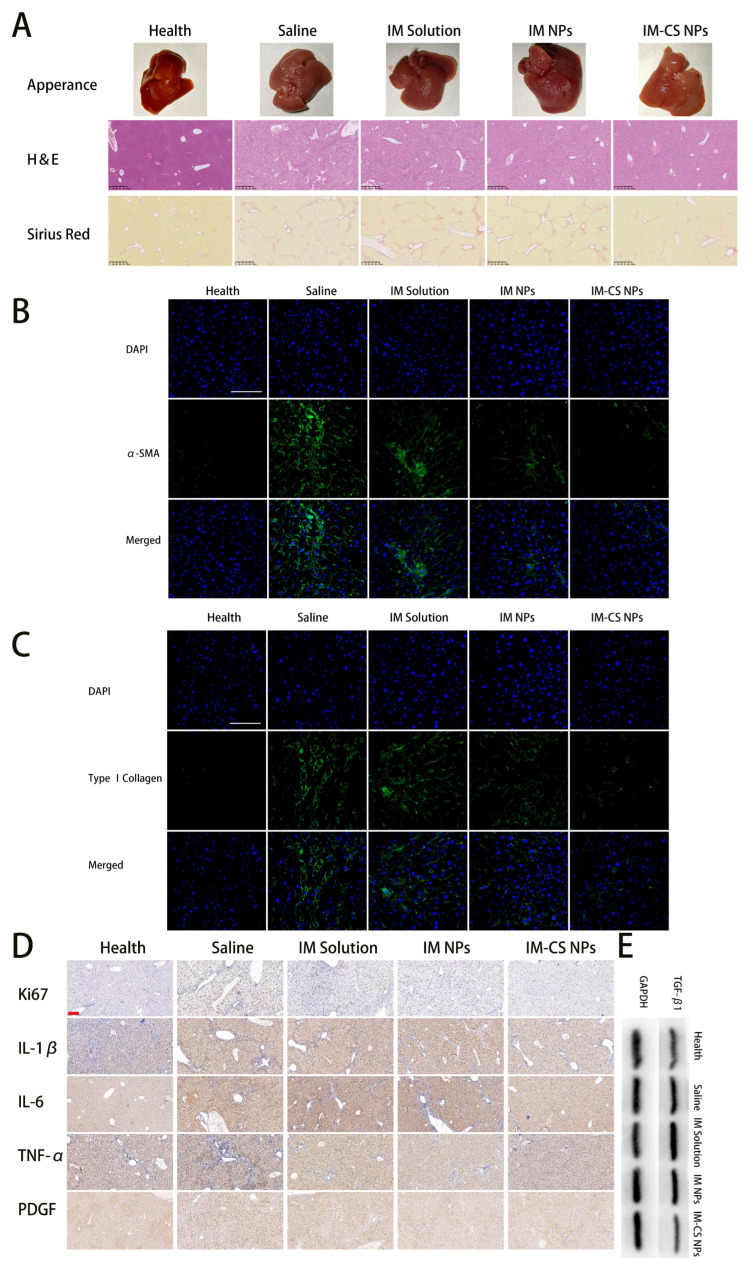
(**A**) Representative photos of liver appearances and HE and Sirius red stains of liver sections from healthy mice and fibrotic mice treated with saline, IM solution, IM NPs, and IM-CS NPs. The scale bar represents 500 µm. (**B**) α-SMA and (**C**) type I collagen staining. The cell nuclei was stained with DAPI (blue), α-SMA and type I collagen were stained with FITC-labeled anti-mouse IgG (green). The scale bars represent 100 µm. (**D**) Representative photos of Ki67, IL-1β, IL-6, TNF-α, and PDGF immunohistochemistry of liver sections from healthy mice and fibrotic mice treated with saline, IM solution, IM NPs, and IM-CS NPs. The scale bar represents 100 µm. (**E**) Western blot analysis of TGF-β1 levels in liver tissues. GAPDH was included as a loading control.

Liver fibrosis mice were administered with different preparations for four weeks. In Figure 4A, the appearance of visible particles on the surfaces of the livers proves that the livers had been severely damaged. H&E and Sirius Red stains showed that the livers of the mice in the saline group had many collagens, ECM, and obvious inflammatory cell infiltration, proving that significant liver fibrosis occurred. Although the liver fibrosis that was treated with the IM solution or IM NPs gradually eased, the mice’s liver surfaces still had some rough particles after the treatment, and the H&E and Sirius Red stains also proved that the liver fibrosis of the mice was still very serious. The above results indicate that the IM solution and IM NPs have only a limited effect on liver fibrosis in mice. However, the livers of the IM-CS NP-treated group showed a smooth surface like the livers of healthy mice, with no rough particles. The results of the H&E and Sirius Red stains also showed that there was only a little collagen and ECM deposition, which strongly proved that IM-CS NPs can exert a good anti-liver fibrosis effect. Semi-quantitative analysis of the positive rate of Sirius scarlet staining also yielded results showing the same trend (Figure S5A). After the liver develops fibrosis, it secretes a large amount of ECM, and this causes the liver to become heavier [68]. In Figure S5B, compared to the healthy group, the liver weight index was elevated after treatment with saline. In several other formulation treatment groups, the liver weight index decreased to varying degrees, and the liver weight index of the IM-CS NP group decreased the most obviously and was close to that of the healthy group.

Many biochemical indicators in the serum reflect the severity of liver fibrosis [69,70]. In liver fibrosis mice, serum levels of (Figure S5C) ALT and (Figure S5D) AST were substantially elevated. Compared with the saline-treated group, ALT and AST were lower in the IM-CS NP-treated group and were closest to the levels found in normal mice.

When liver fibrosis occurs, quiescent HSCs are activated and secrete large amounts of ECM, aHSCs specifically express α-SMA protein, and ECM is dominated by type I collagen [11,71]. As shown in Figure 4B, the healthy group had no α-SMA expression. However, the saline group had significant expression of α-SMA, indicating that a massive number of HSCs were activated. The number of aHSCs in the IM-CS NP-treated group was less than that in the other three modeling groups; stains of (Figure 4C) type I collagen also yielded results showing the same trend. In conclusion, IM-CS NPs could inhibit the activation of HSCs and exert a significant anti-liver fibrosis effect.

During the development of liver fibrosis, there is proliferation and infiltration of inflammatory cells and secretion of various inflammatory factors. We evaluated the following indicators by immunohistochemical staining to evaluate the degree of inflammation during liver fibrosis: IL-1β, IL-6, Antigen Ki67, and TNF-α. Figure 4D shows that there were a lot of brown-yellow precipitates in the saline group, while after the IM solution, IM NPs, and IM-CS NPs were administered, the brown-yellow precipitates were reduced. Moreover, the mice treated with IM-CS NPs had the lowest inflammatory response in the liver tissue. The results are similar to the H&E staining results. They show that IM-CS NPs have a better effect in inhibiting inflammatory response, thereby inhibiting the development of liver fibrosis.

The cytokines TGF-β1 and PDGF play a very important role in the activation of HSCs. We used immunohistochemical staining to characterize the levels of (Figure 4D) PDGF and WB to characterize the levels of (Figure 4E) TGF-β1 in each group. The TGF-β1 grayscale values and PDGF positivity in the saline group were much greater than those in the healthy group, while after treatment with IM-CS NPs, the levels of TGF-β1 and PDGF were reduced, indicating that IM-CS NPs can reduce the activation of HSCs by inhibiting the secretion of TGF-β1 and PGDF, thus exerting good anti-liver fibrosis efficacy.

### 3.14. IM-CS NPs Have Good Safety

**Figure 5:**
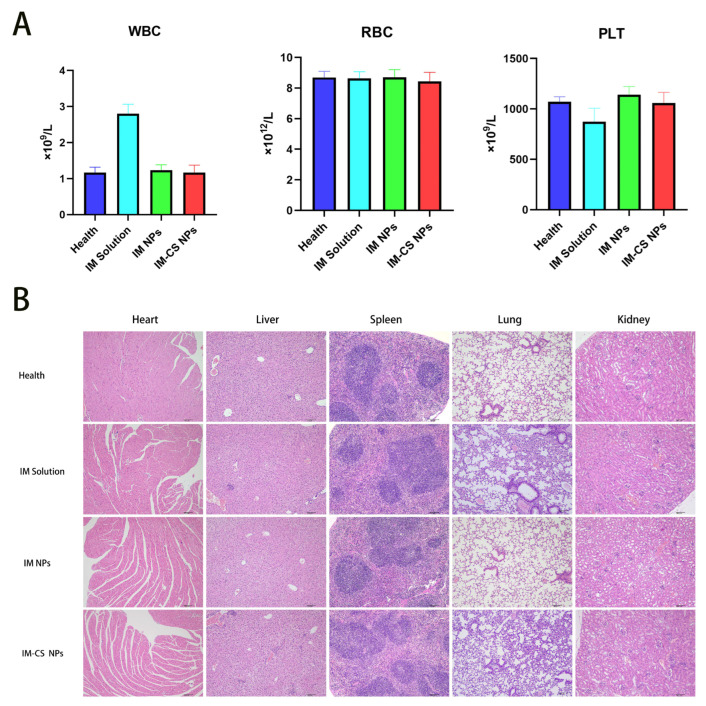
(**A**) WBC, RBC, and PLT counts of healthy mice treated with saline, IM solution, IM NPs, and IM-CS NPs. Data represent means ± SDs (n = 5). (**B**) Histological observation (H&E staining) of major organs treated with saline, IM solution, IM NPs, and IM-CS NPs. The scale bar represents 100 µm.

The safety of targeted drug delivery systems is an important factor in their ability to be developed and applied, and examining the safety of IM-CS NPs is critical to evaluating their value for clinical application. Routine blood analysis (Figure 5A) results demonstrated that IM-CS NPs have a high safety profile in mice, and H&E stains demonstrated that IM-CS NPs have no significant toxicity to major organs (Figure 5B).

## 4. Conclusions

In summary, we fabricated IM-loaded nanoparticles for anti-fibrotic therapy based on a novel design and synthesized amphiphilic CS as the carrier to augment the aHSC-targeted drug delivery efficacy and the therapeutic outcome for hepatic fibrosis. The findings of this study corroborate the hypothesis that amphiphilic CS can enhance the encapsulation efficiency of IM, as well as augment the accumulation of drug in aHSCs. This augmentation is attributable to two factors: CD44-mediated endocytosis and the small particle size (85–95 nm) of CS. Additionally, IM-CS NPs exhibited excellent anti-fibrosis ability in vitro by dual inhibition of TGF-β and PDGF signaling pathways, restoring aHSCs to a quiescent state. More importantly, after tail vein injection, IM-CS NPs showed remarkable anti-fibrotic effects in the CCl4-induced mouse model, with minimal imatinib-induced cytotoxicity to organs, including the liver. In a word, our study not only highlights a promising strategy for hepatic fibrosis treatment but also provides a new insight into the development of future anti-fibrotic nano-drug delivery systems.

## Figures and Tables

**Table 1:** Inhibitors and their concentrations.

Inhibitors	MβCD	Dextran Sulfate	Nystatin	Amiloride	Chlorpromazine	Chondroitin Sulfate	CD44 Antibody
Concentrations	10 µM	100 µg/mL	30 µM	20 µM	10 µg/mL	10 mg/mL	1 µM

**Table 2:** Characterization of IM NPs and IM-CS NPs.

Sample	Particle Size (nm)	PDIs	DLC (%)	EE (%)
IM NPs	96.25 ± 2.1	0.267 ± 0.009	6.25 ± 0.2	97.4 ± 0.1
IM-CS NPs	85.42 ± 1.4	0.261 ± 0.012	5.71 ± 0.1	99.8 ± 0.1

Data represent means ± SDs (*n* = 3).

**Table 3:** Pharmacokinetic parameters of IM solution, IM NPs, and IM-CS NPs.

Sample	IM Solution	IM NPs	IM-CS NPs
Dose (mg/kg)	8.0	8.0	8.0
C_max_ (ug/mL)	1.576	2.114 ***	2.807 ****
T_max_ (min)	0.830	0.830	0.830
AUC	2.357	4.820 ****	6.777 ****
t_1/2_ (min)	0.991	2.222 **	2.771 ***

Each value represents the mean ± SD (n = 5). ** *p* < 0.01; *** *p*<0.001; **** *p* < 0.0001 vs. IM solution.

## Data Availability

Data are contained within the article or the Supplementary Materials.

## Supplementary Materials

The following supporting information can be downloaded at https://www.mdpi.com/article/10.3390/pharmaceutics17030351/s1, Figure S1: (A) ^1^H NMR spectra of CS-HDA synthesized by CS:HAD = 1:1. (B) ^1^H NMR spectra of CS-HDA synthesized by CS:HAD = 1:0.5. (C) ^1^H NMR spectra of CS-HDA synthesized by CS:HAD = 1:0.2; Table S1: Effect of the feeding ratio of CS and HDA on the degree of CS-HDA modification; Figure S2: (A) CMC fitting curve of mixed micelles. (B) Cellular uptake of DID-CS (10% proportion) NPs, DID-CS (20% proportion) NPs, and DID-CS (30% proportion) NPs after incubation with activated HSCs and HepG_2_ cells, as observed by flow cytometric analysis. Data represent means ± SDs (n = 3). **** *p* < 0.0001. (C) The average fluorescence intensity of liver fibrosis model mice at 2, 6, 12, and 24 h after injection of different preparations (means ± SDs, n = 3). * *p* < 0.05; ** *p* < 0.01; *** *p* < 0.001. (D) In vivo DID fluorescence images showing the biodistribution of DID-CS (10% proportion) NPs, DID-CS (20% proportion) NPs, and DID-CS (30% proportion) NPs in liver fibrotic mice at 2, 6, 12, and 24 h after injection; Table S2: CMC value of mixed micelles; Figure S3: (A) HSC-T6 cells stimulated with different concentrations of TGF-β1. (B) Expression of CD44 receptors in different cells. (C) Appearance and histological images of normal and fibrotic livers after H&E, Masson, and Sirius Red staining. The scale bar represents 500 µm; Figure S4: Liver tissue samples from fibrotic mice were sectioned and subjected to immunofluorescence staining using specific antibodies against α-SMA (to identify HSCs), F4/80 (to identify Kupffer cells), CD31 (to identify HSECs), HNF-4α (to identify HPCs), CD44 (to identify CD44 receptors [red]) and DAPI (to identify cell nuclei [blue]).The fluorescent α-SMA, F4/80, CD31, HNF-4α is shown in green. The fluorescent α-SMA, F4/80, CD31, or HNF-4α is shown in green. Co-localization was determined where the green and red fluorescence perfectly merged. The scale bar represents 100 µm; Figure S5: (A) The positive of Sirius scarlet staining, (B) liver mass index, (C) AST levels and (D) ALT levels of healthy mice, fibrotic mice treated with saline, fibrotic mice treated with IM solution, fibrotic mice treated with IM NPs, and fibrotic mice treated with IM-CS NPs. Data represent means ± SDs (n = 6).

## Abbreviations

The following abbreviations are used in this manuscript:

aHSCs	Activated hepatic stellate cells
ALT	Alanine transaminase
AST	Aspartate transaminase
HPCs	Hepatic parenchymal cells
ECM	Extracellular matrix
TGF-β	Transforming growth factor beta
PDGF	Platelet-derived growth factor
IL-1β	Interleukin 1 beta
IL-6	Interleukin-6
TGF-β1	Transforming growth factor-β1
TNF-α	Tumor Necrosis Factor-α
HSECs	Hepatic sinusoidal endothelial cells
IM	Imatinib
TDDS	Targeting drug delivery system
CS	Chondroitin sulfate
CS-HDA	Chondroitin sulfate-1-hexadecylamine
PDIs	Polydispersive index
DLC	Drug loading capacity
EE	Entrapment efficiency
