# Supplementary material for: Chondroitin Sulfate-Based Imatinib Nanoparticles Targeting Activated Hepatic Stellate Cells Against Hepatic Fibrosis
Source: Pharmaceutics. 2025 Mar 9;17(3):351. doi: 10.3390/pharmaceutics17030351 (PMC11944399; doi:10.3390/pharmaceutics17030351)
Supplement: Supplementary file 1 [file pharmaceutics-17-00351-s001.zip › pharmaceutics-3438650-supplementary.pdf]

Supporting Information

Chondroitin Sulfate-Based Imatinib Nanoparticles Targeting Activated Hepatic Stellate Cells Against Hepatic Fibrosis

Xunzhi Liu [1,†], Changlong Fang [2,†], Hongling Yu [1], Lu Huang [1], Jiaxing Feng [1], Shiqin Luo [1], Li Song [3], Mengying Wu [1], Yulu Tan [1], Jianxia Dong [4], Tao Gong [1] and Peihong Xiao [3,*]

[1] Key Laboratory of Drug-Targeting and Drug Delivery System of the Education Ministry and Sichuan Province, Sichuan Engineering Laboratory for Plant-Sourced Drug and Sichuan Research Center for Drug Precision Industrial Technology, West China School of Pharmacy, Sichuan University, Chengdu 610041, China; liuxunzhi00@163.com (X.L.); 17844613439@163.com (H.Y.); huanglu66666@126.com (L.H.); f10174844487@163.com (J.F.); luoshiqin1999@163.com (S.L.); wumengying15@163.com (M.W.); yulutan_scu_edu@163.com (Y.T.); gongtaoy@126.com (T.G.)

[2] Department of Pharmacy, Chongqing University Fuling Hospital, Chongqing University, Chongqing 408099, China; fangchanglong12@163.com

[3] Department of Laboratory Medicine and Sichuan Provincial Key Laboratory for Human Disease Gene Study, Sichuan Provincial People's Hospital, School of Medicine, University of Electronic Science and Technology of China, Chengdu 610041, China; 202421130234@std.uestc.edu.cn

[4] Department of Pharmacy, West China Hospital, Sichuan University, Chengdu 610041, China; 15802891207@163.com

* Correspondence: peihongxiao@uestc.edu.cn

† These authors contributed equally to this work.

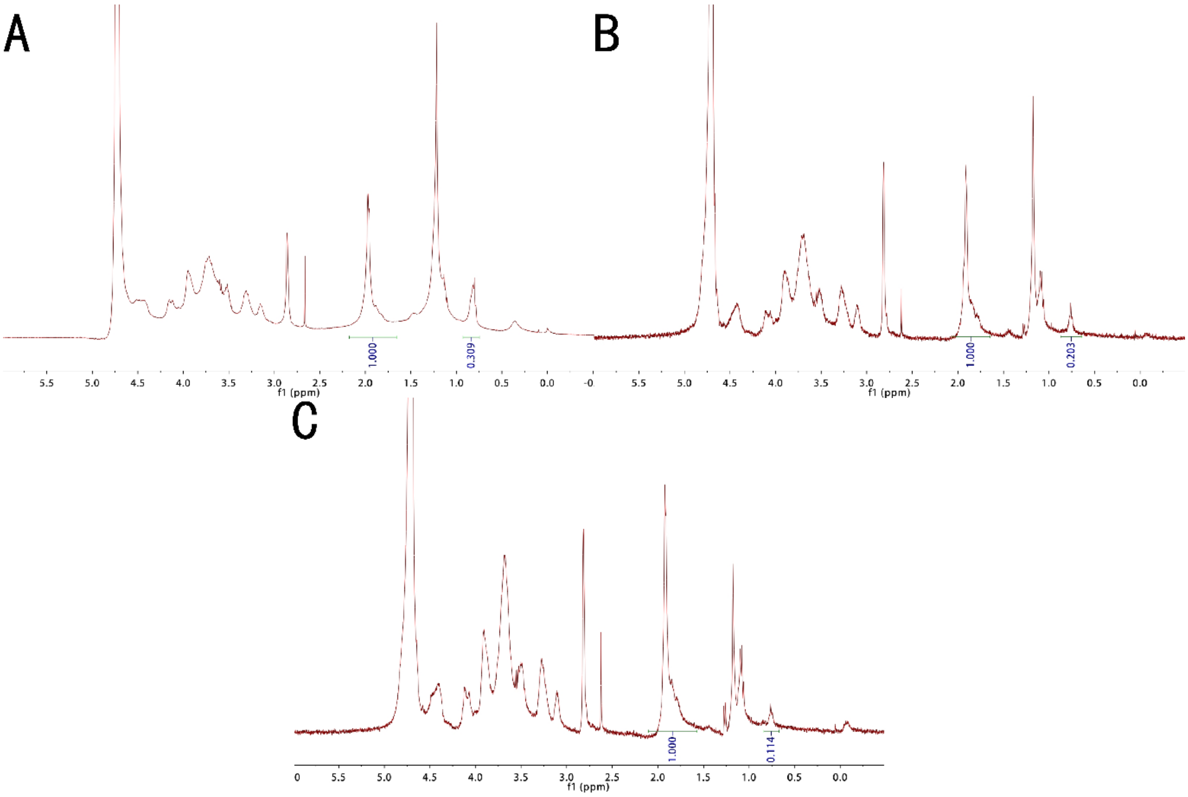

Figure S1. (A) ^{1}H NMR spectra of CS-HDA synthesized by CS : HAD = 1 : 1 (B) ^{1}H NMR spectra of CS-HDA synthesized by CS : HAD = 1 : 0.5 (C) ^{1}H NMR spectra of CS-HDA synthesized by CS : HAD = 1 : 0.2

Table S1. Effect of the feeding ratio of CS and HDA on the degree of CS-HDA modification.

CS: HDA	Modified degree（%)
1:0.2	11.4
1:0.5	20.3
1:1	30.9

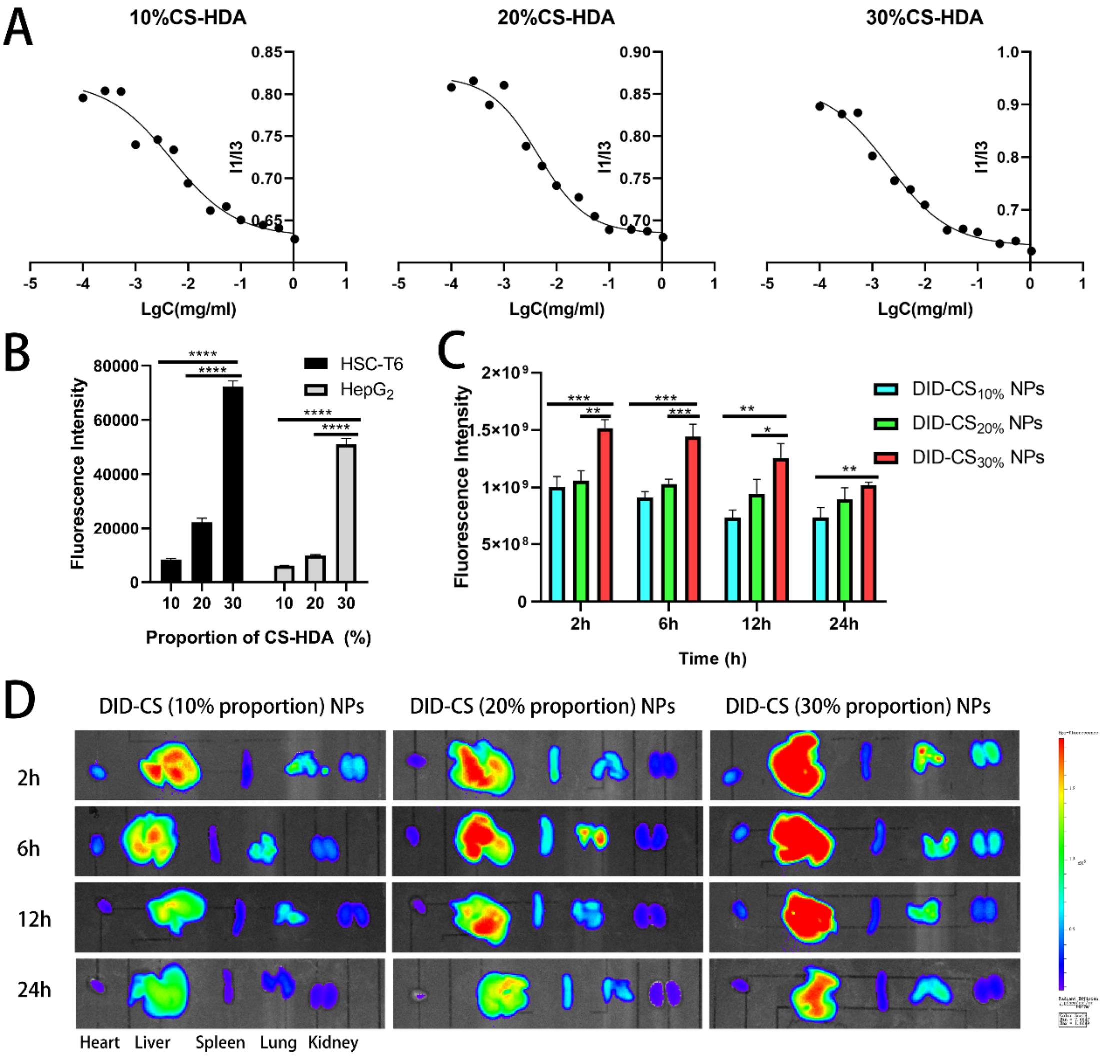

Figure S2. (A) CMC fitting curve of mixed micelles. (B) Cellular uptake of DID-CS (10% proportion) NPs, DID-CS (20% proportion) NPs and DID-CS (30% proportion) NPs after incubation with activated HSCs and $HepG_2$ cells; observed by flow cytometric analysis. Data represent mean ± SD (n = 3). $^{****}P < 0.0001$. (C) The average fluorescence intensity of liver fibrosis model mice at 2, 6, 12, and 24 h after injection of different preparations (mean ± SD, n = 3). $^{*}P < 0.05$; $^{**}P < 0.01$; $^{***}P < 0.001$. (D) In vivo DID fluorescence images showing the bio-distribution of DID-CS (10% proportion) NPs, DID-CS (20% proportion) NPs and DID-CS (30% proportion) NPs in liver fibrotic mice at 2, 6, 12, and 24 h after injection.

Table S2. CMC value of mixed micelles.

Proportion of CS-HDA (%)	10	20	30
CMC (mg/mL)	4.82×10^{-3}	4.47×10^{-3}	2.04×10^{-3}

Data represent mean ± SD (n = 3).

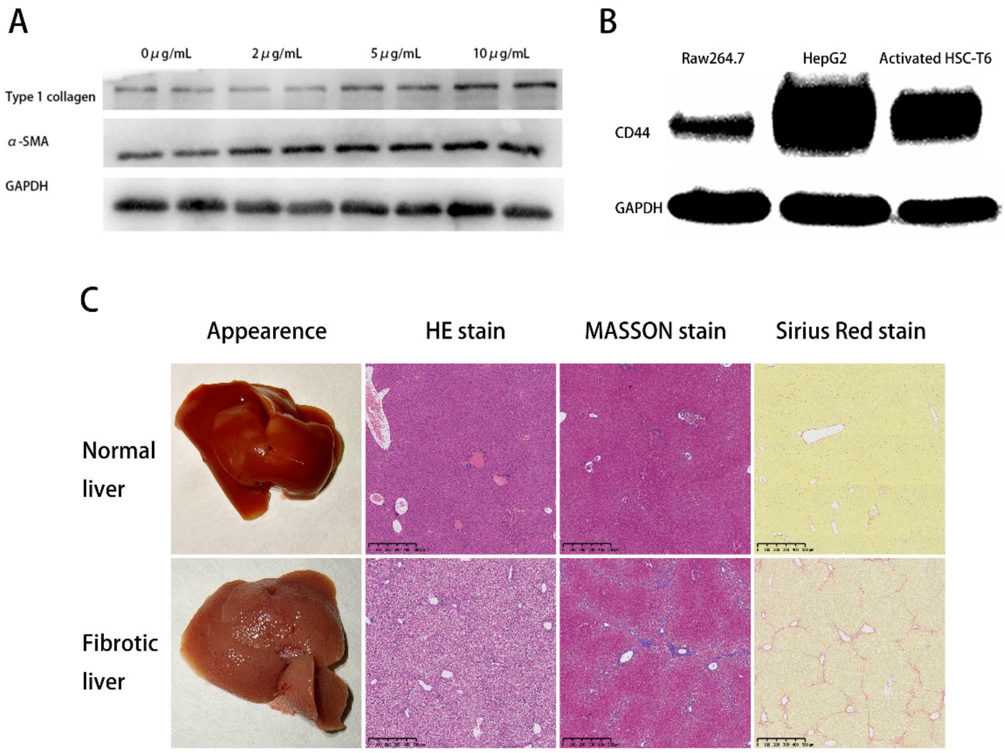

Figure S3. (A) Different concentrations of TGF-β1 stimulate HSC-T6 cells. (B) Expression of CD44 receptors in different cells. (C) Appearance and histological images of normal and fibrotic livers after H&E, Masson and Sirius Red stain. The scale bar represents 500 μm.

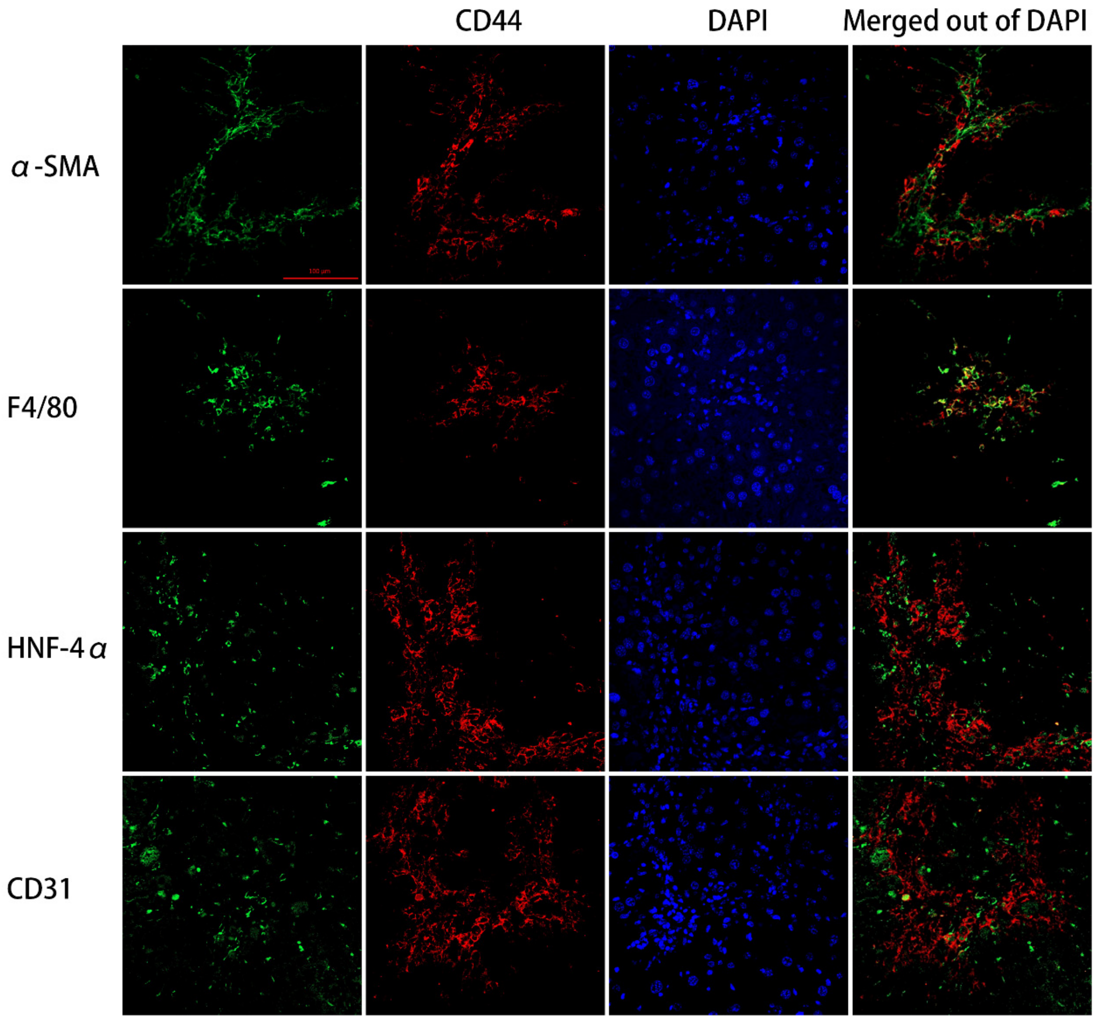

Figure S4. The liver tissue from liver fibrotic mice was then sectioned and immunofluorescent stained by α-SMA（for HSCs）, F4/80（for Kupffer cells）, CD31（for HSECs）or HNF-4α（for HPCs）, CD44 for CD44 receptors (red) and DAPI for cell nucleus (blue). The fluorescent α-SMA, F4/80, CD31 or HNF-4α is shown in green. Co-localization was determined where the green and red fluorescence perfectly merged. The scale bar represents 100 μm.

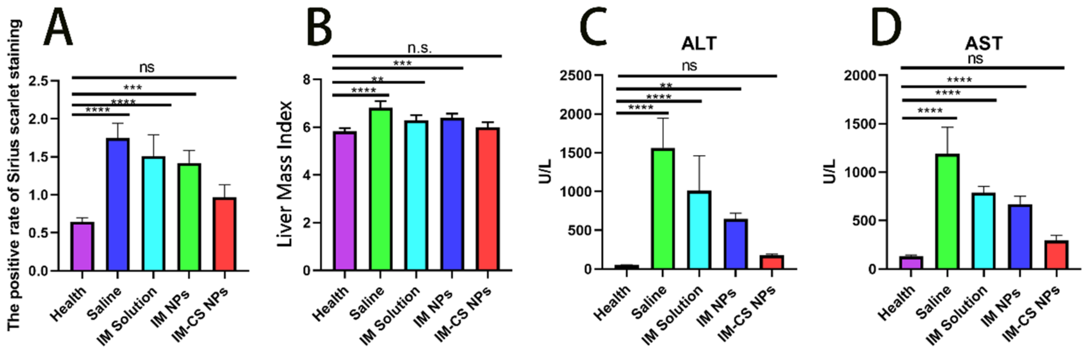

Figure S5. (A) The positive of Sirius scarlet staining, (B) liver mass index, (C) AST and (D) ALT levels of health mice and fibrotic mice treated with saline, IM Solution, IM NPs and IM-CS NPs. Data represent mean ± SD (n = 6).
